# TLR2-induced surface mobilization and release of CD14 in human platelets

**DOI:** 10.1038/s41598-025-22715-7

**Published:** 2025-10-13

**Authors:** Anna Kobsar, Daniela Simao Vaz, Julia Zeller-Hahn, Angela Koessler, Katja Weber, Sabine Kuhn, Christian Stigloher, Juergen Koessler

**Affiliations:** 1 Clinical Transfusion Medicine and Haemotherapy, University Clinic of Wuerzburg, Oberduerrbacher Straße 6, 97080 Wuerzburg, Germany; 2 Imaging Core Facility of the Biocenter, Theodor-Boveri-Institute, University of Wuerzburg, 97074 Wuerzburg, Germany

**Keywords:** Super-resolution microscopy, Toll-like receptors, Protein translocation, Cellular imaging, Imaging the immune system, Innate immune cells

## Abstract

In addition to hemostasis, platelets are involved in immunological processes and express toll-like receptors (TLR) like TLR2 and TLR4. Since CD14 represents an essential co-receptor for TLR in immune cells, this study investigated the presence, the release and the distribution of CD14 in human platelets. Washed human platelets (WP) were used for analysis, either unstimulated or stimulated with the TLR2 agonist Pam3CSK4 or with the TLR4 agonist LPS (lipopolysaccharides from *Escherichia coli*). CD14 expression was detected by flow cytometry, soluble CD14 (sCD14) by an immunoassay. The platelet content of CD14 was measured with Western Blot analysis. Visualization of CD14 was performed with transmission electron microscopy or high-resolution fluorescence microscopy. CD14 expression was detectable after TLR2- or collagen-induced stimulation, but not in resting and TLR4-stimulated platelets. TLR2 activation induced the release of sCD14. In adherent non-permeabilized WP, CD14 expression was found locally condensed in the central region. In permeabilized platelets, an intracellular CD14-specific thread-like staining was visible predominantly along the membrane. CD14 showed a distribution pattern in co-localization with TLR2 and TLR4. In conclusion, platelets contain CD14 molecules co-localized with TLR2 and TLR4, which can be recruited to the surface after specific platelet stimulation and released as sCD14.

**Supplementary Information:**

The online version contains supplementary material available at 10.1038/s41598-025-22715-7.

## Introduction

Platelets are anucleated cell fragments released from megakaryocytes in the bone marrow, appearing in the peripheral blood in concentrations of 150,000 to 400,000 per µL and playing a mandatory role in cellular hemostasis and thrombus formation^1^. In medicine, the inhibition of platelet function is an important pharmacological principle to prevent platelet aggregation and consecutive vessel occlusion^2^. Thrombocytopenia, as a reduction of platelets in the blood, commonly occurs during cancer treatment with radio-chemotherapy or in hematological diseases, accompanied with an increased risk of bleeding and the need for substitution by transfusion of platelet concentrates^3^. Beyond hemostasis, platelets contribute to wound healing or to inflammatory processes and show characteristics of immune cells^4^. For example, they contain a huge number of intracellular chemokines like platelet factor 4 (PF4), platelet activating factor (PAF) or platelet-derived growth factor (PDGF), which are stored in α-granules and released into the surrounding blood or tissue after platelet activation. Similar to immune cells, platelets are also equipped with different subtypes of toll-like receptors (TLR)^4–8^, among them the two most abundant types TLR2 and TLR4^4^. TLR are engaged in the recognition of pathogen- or damage-associated molecular patterns, so-called PAMPs or DAMPs, and activated via the MyD88-dependent pathway^9^. TLR2, forming heterodimers with TLR1 or TLR6, can be stimulated with acylated lipoproteins like the synthetic triacylated molecule Pam3CSK4^10^. Lipopolysaccharides (LPS), e.g. from the gram-negative bacteria *Escherichia coli*, are ligands for TLR4^11^. In immune cells, both receptor types are associated with the glycosyl-phosphatidylinositol–anchored protein CD14^12–14^, as a common co-receptor and as a prerequisite for ligand binding and prompting rapid signaling^12–15^.

Recently, we elucidated that functional effects are mediated via stimulation of TLR2 and TLR4 in washed human platelets (WP)^16,17^. TLR2 activation led to facilitation of platelet aggregation, to the uptake of ligands, to reactive oxygen species (ROS) production or to platelet adhesion. TLR4 activation is able to induce platelet-leukocyte interactions leading to formation of platelet-neutrophil complexes^16,17^. In immune cells, CD14 exists in two forms, as a membrane-bound form (via glycosylphosphatidylinositol anchor; long isoform) and as a soluble form (soluble CD14, sCD14; short isoform)^18,19^. However, up to now, it remained unclear if resting WP bear CD14 on their membrane surface^18,19^. In addition, CD14 may also be recruited from intracellular pools to the surface of platelets, thereby providing their co-receptor function and enabling TLR2 or TLR4 ligand binding.

For clarification, this study addressed the presence and the distribution of CD14 in resting and stimulated WP. The analytical procedures comprised the detection of CD14 expression by flow cytometry, the measurement of sCD14 using an immunoassay, the measurement of CD14 in platelet pellets with Western Blot analysis and the visualization of CD14 with electron microscopy or high-resolution immunofluorescence microscopy. WP were used for analysis to rule out plasma as an external source for CD14 molecules.

The results of this study provide evidence that platelets contain CD14 molecules intracellularly, located along the platelet membrane. Upon specific stimulation via TLR2 and collagen, but not via TLR4, CD14 is expressed on the surface membrane and partially released as short, soluble isoform (sCD14). After adhesion to collagen-coated slides, platelets show CD14 expression in a locally condensed area in the central region of activated platelets. Immunofluorescence microscopy shows co-localization of CD14 with TLR2 and TLR4.

## Results

### Stimulation of TLR2, but not of TLR4, induces platelet CD14 expression

**Fig. 1:**
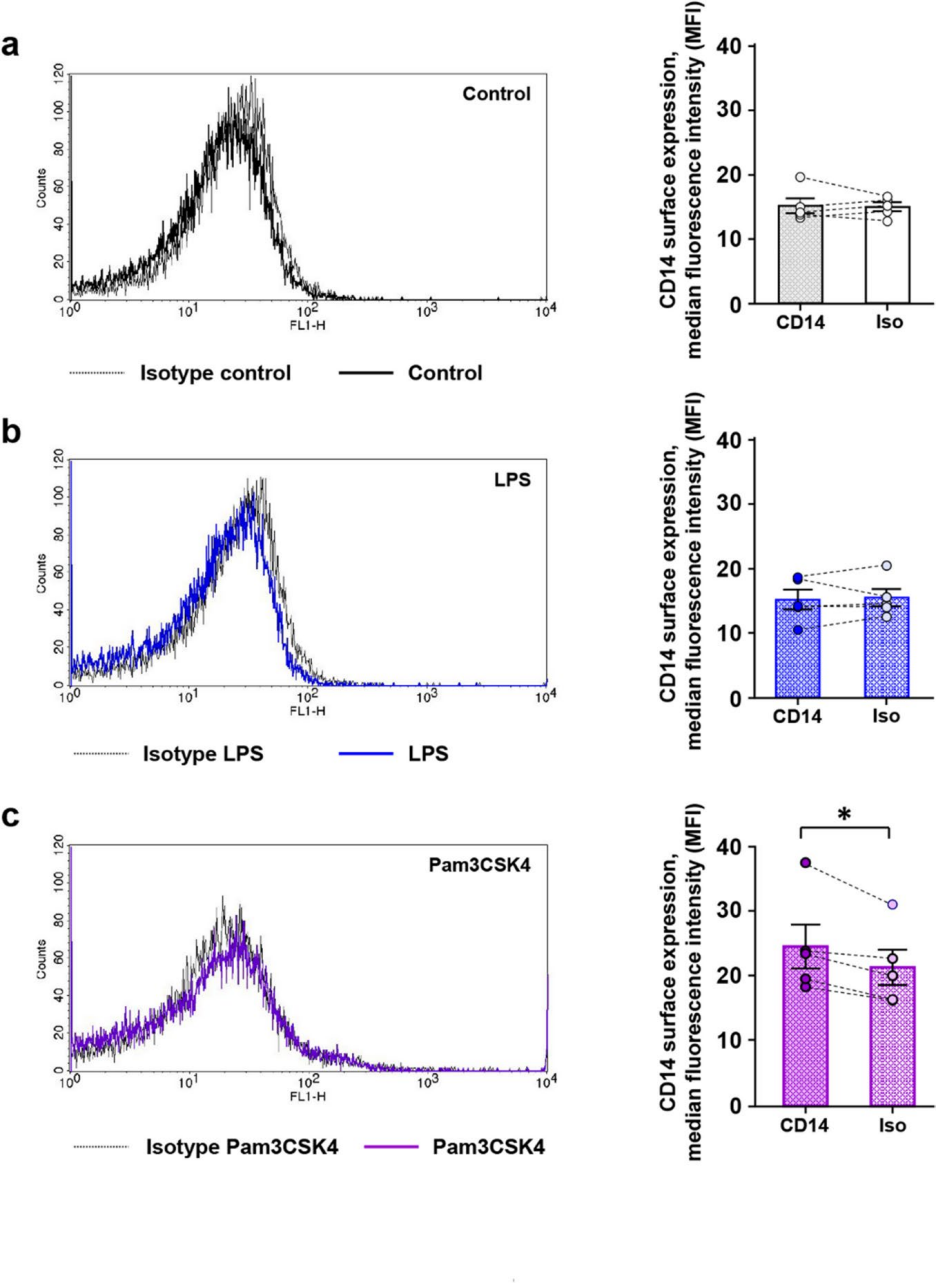
Stimulation with a TLR2 agonist induces CD14 expression in WP. WP (3 × 10^8^ platelets/mL) were incubated with buffer as control (**a**), with 15 µg/mL LPS (**b**) or with 15 µg/mL Pam3CSK4 (**c**), followed by fluorescent staining with an anti-CD14 antibody or with an isotype IgG. CD14 expression measured by flow cytometry is given as median fluorescence intensity (MFI) in arbitrary units. Representative histograms of flow cytometry are shown on the left side, illustrating the absence (**a, b**) or the shift of fluorescence to the right (**c**). In histograms on the right side, results for CD14 expression are presented as mean of median fluorescence intensity values ± SEM; *n* = 5; *p < 0.05.

Platelet surface expression of CD14 was measured by flow cytometry in resting platelets and after stimulation with bacterial LPS or with the lipoprotein Pam3CSK4 (Fig. 1). Resting WP did not express CD14, demonstrated by similar median fluorescence values (MFI) for anti-CD14 antibody staining with 15.2 ± 1.1 MFI and for isotype control with 15.1 ± 1.1 MFI (Fig. 1a). LPS did not induce CD14 expression, indicated by 15.2 ± 1.5 MFI, comparable with the isotype control, indicated by 15.5 ± 1.4 MFI (Fig. 1b). Instead, stimulation with Pam3CSK4 provoked CD14 expression with values of 24.8 ± 3.4 MFI, compared to the isotype control with 21.6 ± 2.7 MFI (Fig. 1c).

### Pam3CSK4, but not LPS, induces the release of sCD14 from WP

**Fig. 2:**
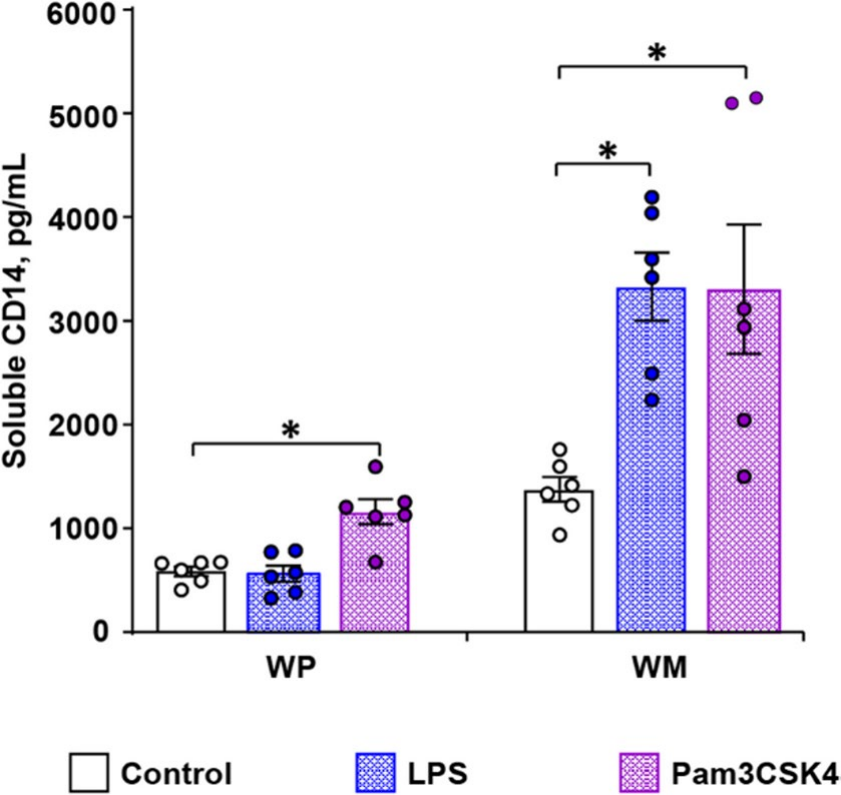
Stimulation of TLR2 in WP induces the release of sCD14 ligand. The levels of released sCD14 were measured in the supernatants of human WP (3 × 10^8^ platelets/mL) stimulated with buffer (control), with 15 µg/mL LPS or with 15 µg/mL Pam3CSK4 for 30 min. WM served as positive controls. Results are presented as mean ± SEM; *n* = 6; *p < 0.05.

The levels of sCD14 secretion were measured with an immunoassay kit in the supernatant of platelet suspensions. For this purpose, WP or washed human monocytes (WM) were incubated with HEPES buffer (as control), 15 µg/mL LPS or 15 µg/L Pam3CSK4 for 30 min at 37 °C (Fig. 2). WP in control samples and LPS-stimulated WP released similar amounts of sCD14, reaching values of 574.5 ± 49.9 pg/mL and 559.5 ± 85.2 pg/mL, respectively. In contrast, Pam3CSK4 induced an increase of sCD14 levels to 1161.0 ± 132.3 pg/mL (Fig. 2). For comparison, sCD14 was also quantified in the supernatant of WM suspensions, prepared from the same donors (Fig. 2). The stimulation with both LPS and Pam3CSK4 led to an emphasized CD14 release from 1371.4 ± 88.7 pg/mL to 3331.2 ± 358.7 pg/mL and to 3288.4 ± 621.3 pg/mL.

### WP contain both the membrane-bound CD14 isoform and sCD14

**Fig. 3:**
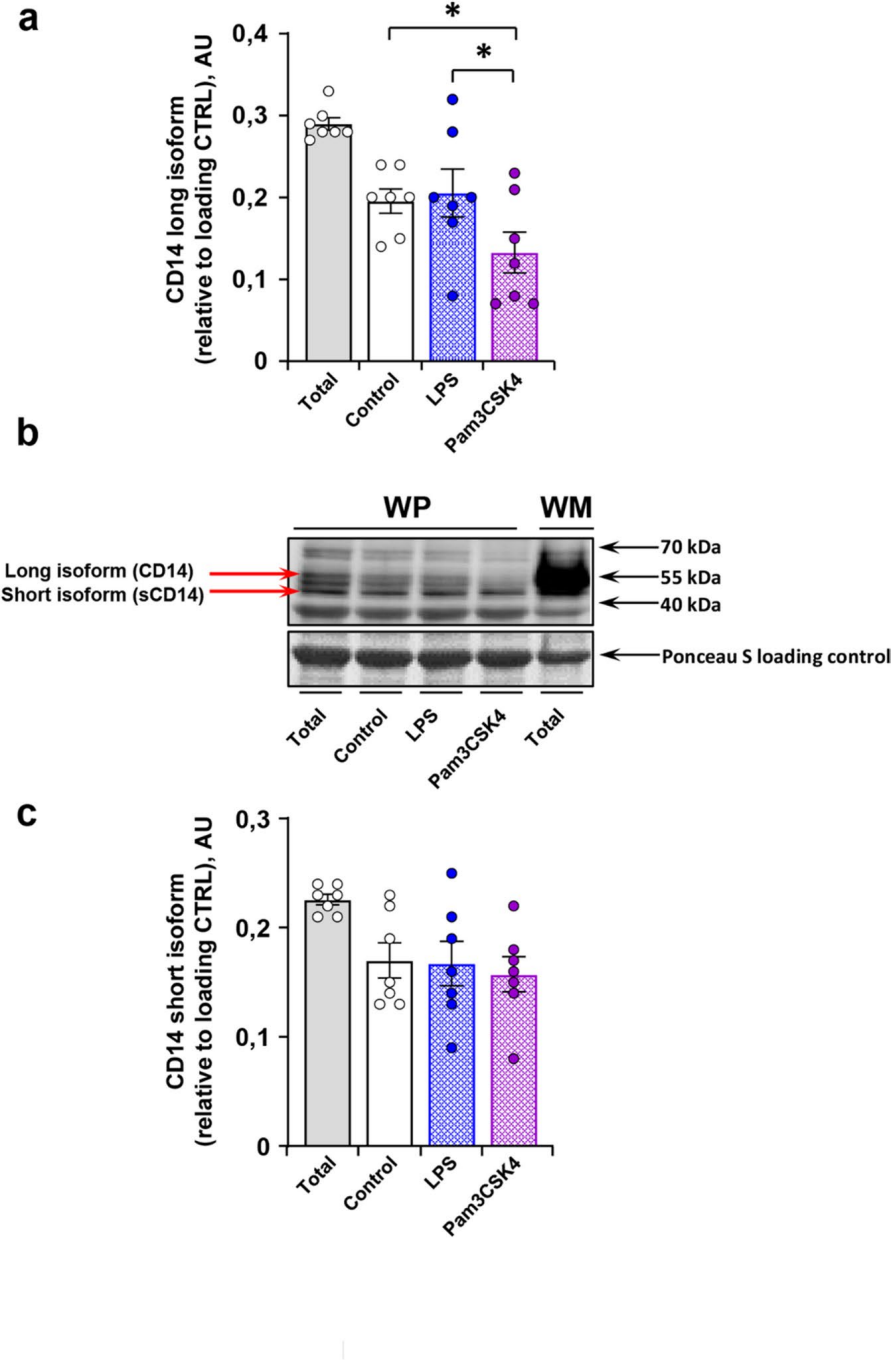
Membrane-bound long isoform of CD14 decreases in TLR2-stimulated WP. Using Western Blot analysis, the amount of membrane-bound CD14 isoforms, long (**a**) and short (**c**), were detected in lysed pellets of human WP (3 × 10^8^ platelets/mL) after incubation with buffer (basal), with 15 µg/mL LPS or with 15 µg/mL Pam3CSK4 for 30 min. WM served as positive controls. A representative Western blot figure is shown (**b**). Ponceau S staining was used as loading control and for normalization. Results are given as mean ± SEM in arbitrary units relative to the loading controls; *n* = 7; *p < 0.05. Original uncropped blots are shown in supplemental figure S1.

Western blot analysis was performed to quantify the content of CD14 in platelets using pellets remaining after removal of supernatants from WP suspensions upon stimulation with LPS or Pam3CSK4 (Fig. 3). WP contained CD14, although much less compared to WM (Fig. 3b). Both known CD14 isoforms—the long, membrane-bound isoform (Fig. 3a) and the short, soluble isoform (Fig. 3c)—are detectable in human WP. Platelet stimulation with Pam3CSK4 reduced the amount of the membrane-bound CD14 isoform in pellets from 0.20 ± 0.02 UA to 0.13 ± 0.03 UA, whereas LPS did not have an influence (Fig. 3a). The content of the short CD14 isoform remained unchanged after stimulation with both LPS and Pam3CSK4 (Fig. 3c).

### CD14 is localized inside of platelets

**Fig. 4:**
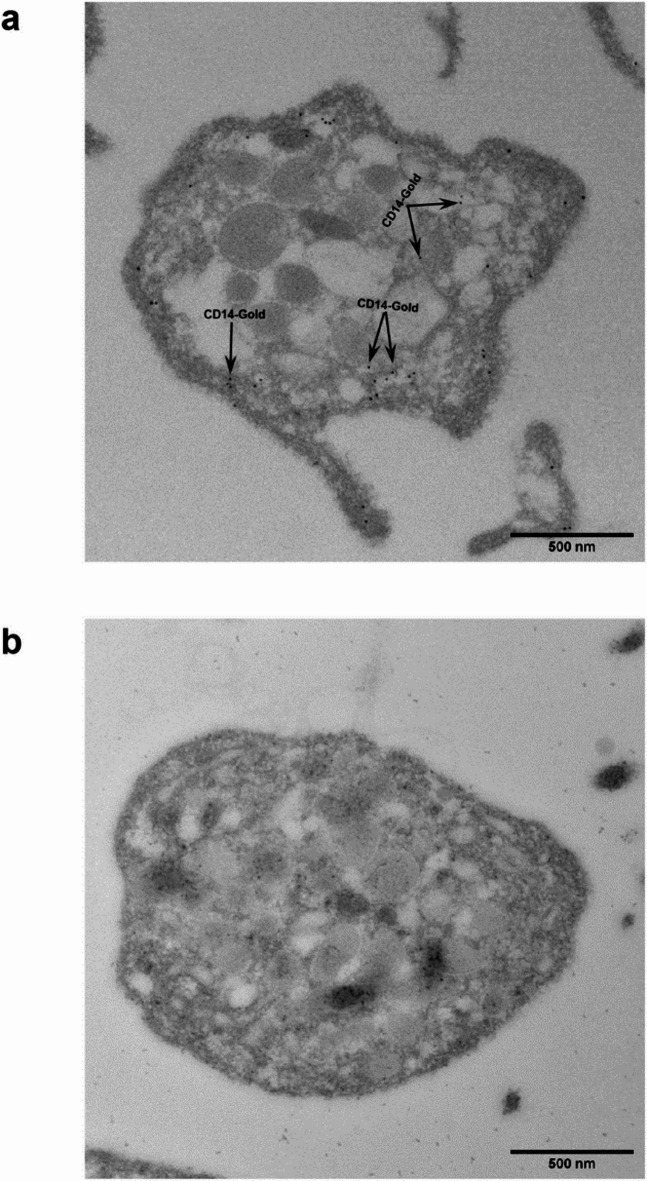
CD14 is located inside of unstimulated, not adherent platelets. The figures show ultrathin sections of human WP using transmission electron microscopy. WP were fixed floating in the suspension and stained with an anti-CD14 (12 nm gold) antibody (**a**) or with isotype control (**b**). A highly specific intracellular staining of CD14 ligand is visible inside of WP along the plasma membrane (**a**), whereas no staining is visible in the negative control (**b**); *n* = 2.

Transmission electron microscopy was used to visualize the internal distribution of CD14 in platelets (Fig. 4). Quiescent WP, fixed in suspension and stained with a polyclonal anti-CD14 antibody, revealed an intracellular staining, particularly along the platelet membrane (Fig. 4a). Using the isotype control, no staining was visible (Fig. 4b).

### CD14 is locally expressed on the surface of adherent WP

**Fig. 5:**
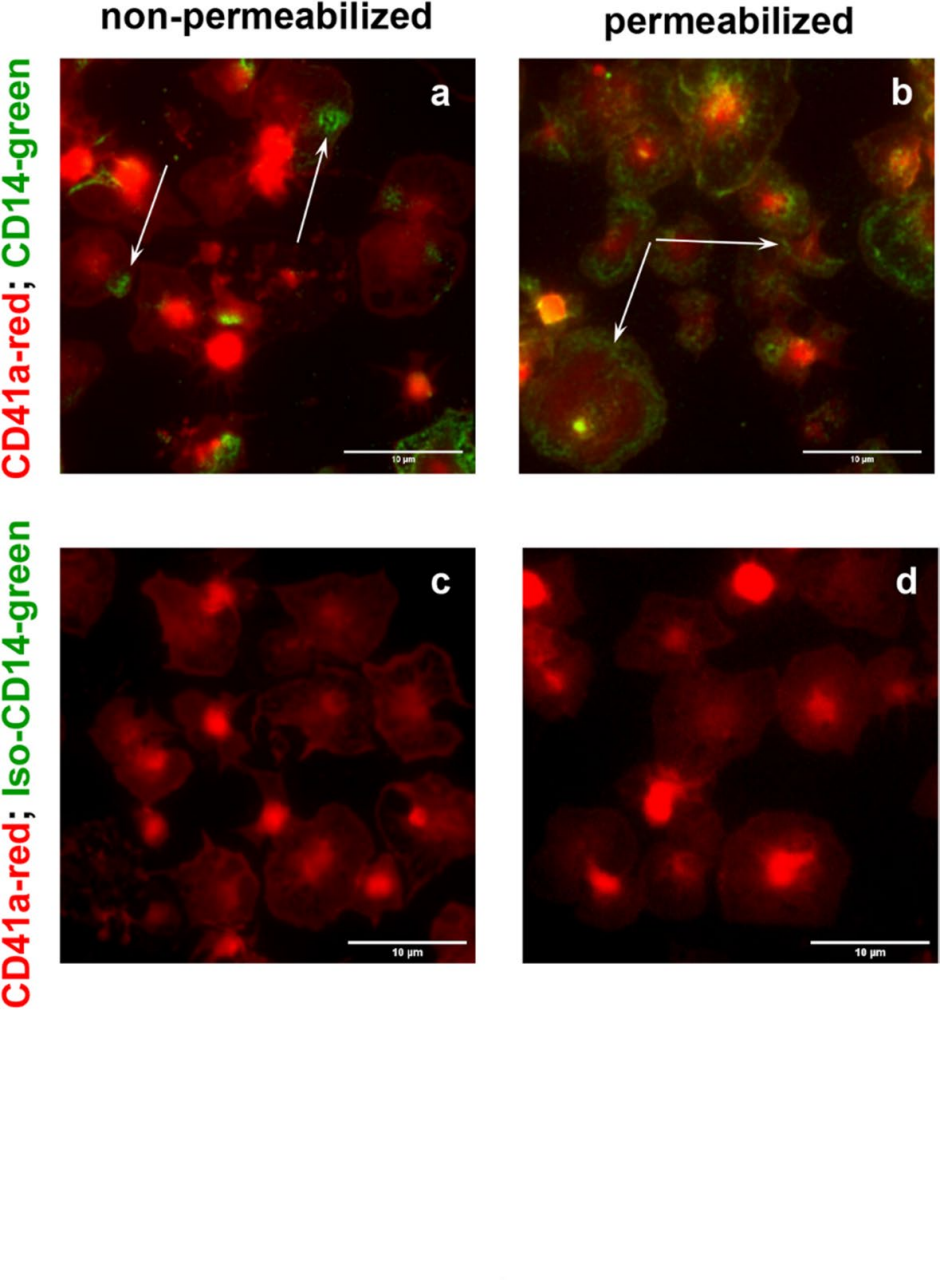
CD14 is expressed on the surface and inside of collagen-adherent platelets. WP (2.5 × 10^7^ platelets/mL), diluted with HEPES and supplemented with 1 mM CaCl2, were seeded onto collagen-coated slides and left to adhere for 30 min at room temperature. After fixation, without (**a,c**) or with permeabilization (**b,d**), platelets were stained overnight with an Alexa Fluor 488-conjugated anti-CD14 antibody and with a Vioblue-conjugated anti-CD41a antibody as indicated, followed by mounting and subsequent analysis on an inverted Nikon Eclipse Ti2 microscope using a 100 x oil immersion objective and a 1.5x zoom (14-bit digitalization). The images show representative slides with adherent unstimulated platelets; *n* = 3.

High-resolution fluorescence microscopy was used to detect the distribution of CD14 on the surface of human WP, adherent to collagen-coated slides (Fig. 5). Staining was performed with human VioBlue-conjugated anti-CD41 antibody (for platelet visualization, here as a virtual red color) and goat anti-CD14 antibody, followed with Alexa FluorTM Plus 488-conjugated donkey anti-goat antibody (specific CD14 visualization, virtual green color). Adherent platelets showed a “fried-egg” morphology, typical for spread platelets as a result of cytoskeletal rearrangements leading to the formation of a small microtubular ring in the central platelet region and to the concentration of cytoskeletal components in the “yolk” spot of the “fried egg”^20^. In non-permeabilized platelets, CD14 was detectable in a locally limited area close to this “yolk” spot (Fig. 5a). In permeabilized platelets, an additional thread-like staining along the membrane was visible (Fig. 5b). There was no visible staining using isotype controls, indicating specific binding of the anti-CD14 antibody (Fig. 5c, d).

**Fig. 6:**
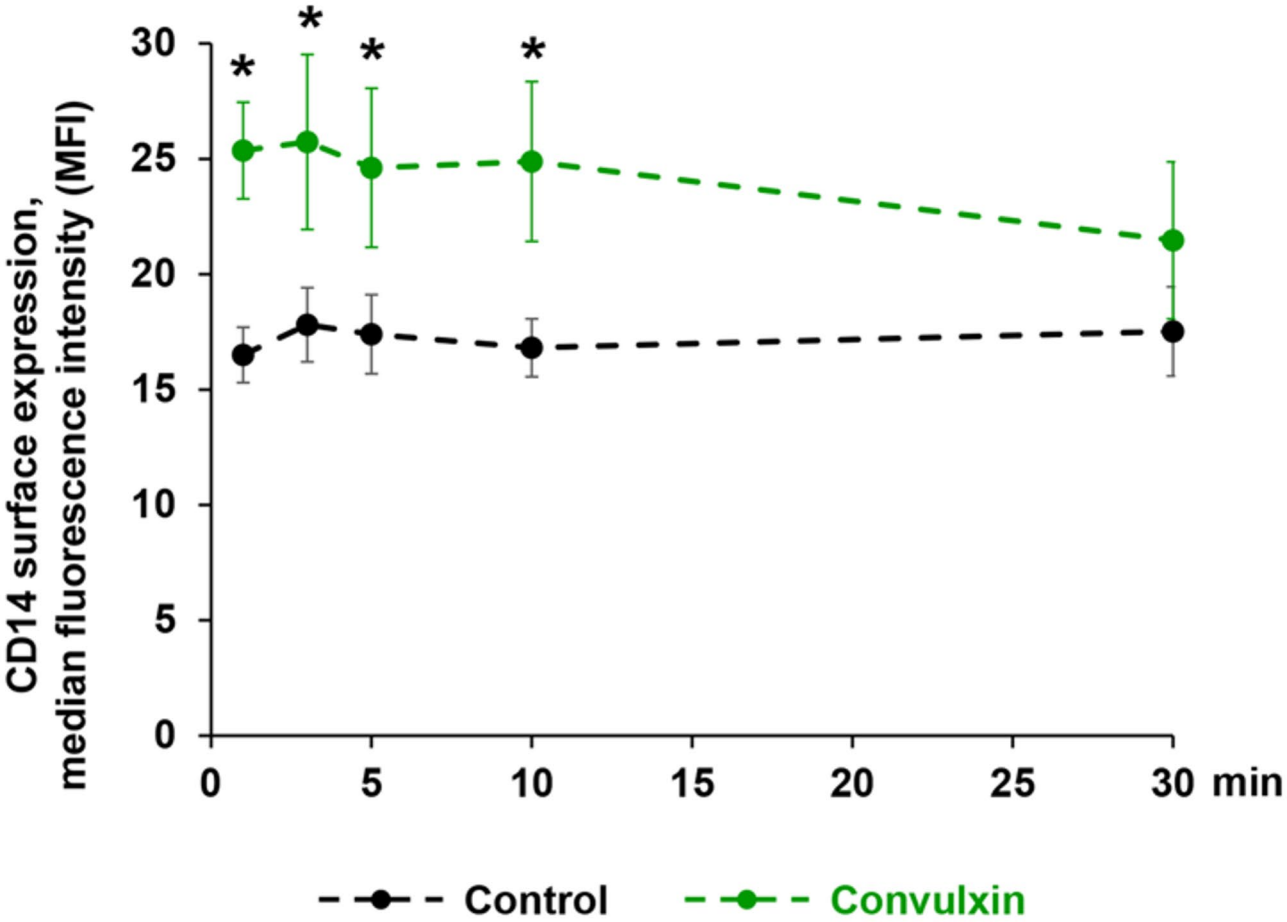
Stimulation with convulxin induces CD14 expression in WP. WP (3 × 10^8^ platelets/mL) were incubated for indicated times with buffer as control or with 100 ng/mL convulxin followed by fluorescent staining with an anti-CD14 antibody or with an isotype IgG antibody, and with Alexa Fluor 488-conjugated donkey anti-goat secondary antibody. Results for CD14 expression measured by flow cytometry are presented as mean of median intensity fluorescence values (MFI) ± SEM; *n* = 6; *p < 0.05 (compared to the corresponding control).

In addition, the time-dependent CD14 expression pattern was analyzed by flow cytometry after stimulation with convulxin, an agonist for the collagen receptor glycoprotein VI (GPVI) (Fig. 6). The results indicated that convulxin is able to induce a transient CD14 expression with an initial increase from 16.5 ± 1.2 MFI in the unstimulated control sample to 25.4 ± 2.1 MFI after 1 min, and to maintained values of 25.7 ± 3.8 MFI after 3 min, 24.6 ± 3.4 MFI after 5 min and 24.9 ± 3.5 MFI after 10 min. Consecutively, convulxin-induced fluorescence decreased to the baseline of unstimulated controls within 30 min.

### CD14 is co-localized with TLR2 and TLR4 in adherent platelets

**Fig. 7:**
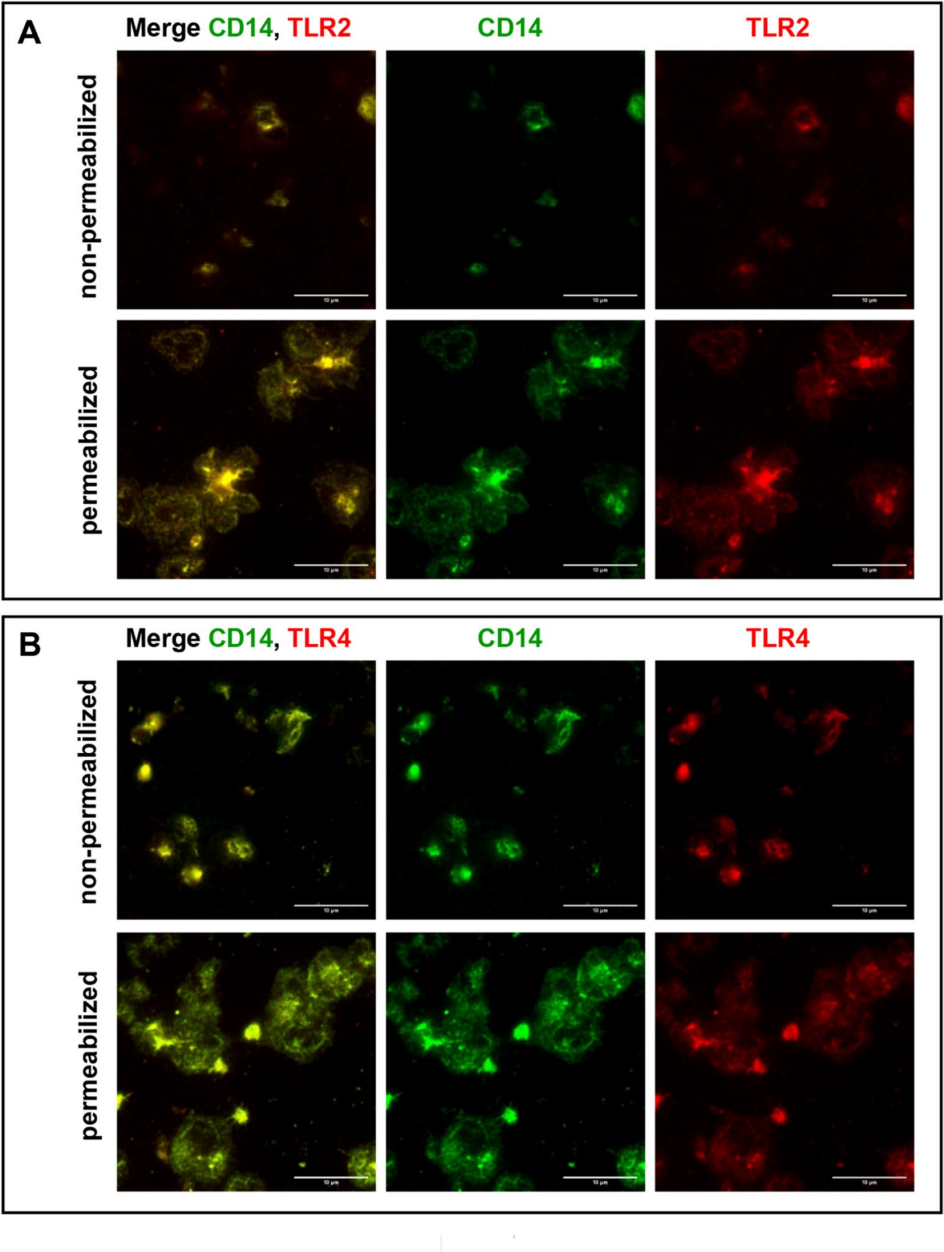
Co-localization of CD14 with TLR2 and TLR4 in adherent platelets. WP (2.5 × 10^7^ platelets/mL), diluted with HEPES and supplemented with 1 mM CaCl_2_, were seeded onto collagen-coated slides and left to adhere for 30 min at room temperature. After fixation, without or with permeabilization, platelets were stained overnight with a goat anti-CD14 antibody and a mouse anti-TLR2 (**A**) or a mouse anti-TLR4 (**B**) antibody as indicated, followed by staining with an Alexa Fluor 488-conjugated donkey anti-goat antibody and a DyLight 550-conjugated donkey anti-mouse antibody, mounting and subsequent analysis on an inverted Nikon Eclipse Ti2 microscope using a 100 x oil immersion objective and a 1.5 x zoom (14-bit digitalization). The images show representative slides with adherent unstimulated platelets; *n* = 5.

The co-localization of CD14 with TLR2 and TLR4 was explored by immunofluorescence microscopy after co-staining of the molecules in adherent platelets on collagen-coated slides (Fig. 7). In non-permeabilized platelets, TLR2 (Fig. 7a) and TLR4 (Fig. 7b) were detectable in an identical pattern compared to staining of CD14 close to the “yolk” spot. Accordingly, in permeabilized platelets, CD14 showed an additional thread-like staining congruent to TLR2 and TLR4.

Further images of immunofluorescence microscopy addressing co-localization in CD41-labelled platelets, and including isotype controls, are shown as supplemental material (Fig. S2-S5).

## Discussion

In peripheral blood, the platelet count exceeds the leukocyte count up to 50-fold^21^, making platelets available at the site of vessel injuries in a high number to encounter invading antigens or DAMPs and PAMPs. TLR2 and TLR4 are widely expressed on the platelet membrane and mediate specific functional responses after ligand binding^4^. The mechanism of TLR activation in platelets, however, is not yet fully understood, since TLR commonly require the co-receptor CD14 for activation^12–14^, which has not been detected on the surface of resting WP so far.

The flow cytometric analysis of unstimulated WP confirmed that resting platelets do not bear CD14 molecules on their surface membrane. The lacking CD14 expression was also observable after stimulation with the TLR4 ligand LPS, as a substance only weakly activating platelets^16,22^. In contrast, CD14 expression is well detectable after stimulation with the TLR2 ligand Pam3CSK4, representing a more potent platelet activator^16,22^. These results indicate that platelets may contain an internal pool of CD14, recruitable to the surface membrane after platelet activation.

For experimentation with platelets, it was important to rule out contamination with leukocytes as a source of CD14. Therefore, only the upper part of PRP was used for analysis. In addition, each PRP sample was tested by automated blood count indicating no measurable levels of leukocytes. In microscopic imaging, no leukocytes were visible, indicating purity of samples.

Proteolytic cleavage of the membrane-bound form of CD14 is a well-known mechanism leading to the release of sCD14 (as short CD14 isoform) from monocytes^23^. The measurement of sCD14 revealed an accumulation of sCD14 in the supernatant of platelet suspensions after Pam3CSK4 or convulxin stimulation. It can be concluded that CD14 molecules are obviously cleaved and consecutively shed into the surrounding medium upon transient surface expression after activation via TLR2 and GPVI.

The additional Western blot analysis confirmed that pellets of resting platelets contained both the long, membrane-bound CD14 and the sCD14 isoform. In general, the content of CD14 is markedly lower in WP compared to classical immune cells like WM. Upon activation via TLR2, the platelet content of membrane-bound CD14 decreased, whereas the content of sCD14 remained unchanged. Platelet stimulation via TLR4 neither interferes with the membrane-bound isoform nor with the sCD14 levels. These results confirm that the long CD14 isoform is transferred to the platelet surface membrane, and consecutively shed into the surrounding medium, whereas the intracellular sCD14 isoform may serve as an initial component for the regeneration of the long CD14 isoform inside of platelets.

In total, this study verifies the presence of CD14 in platelets for the first time, whereas previous reports only indirectly indicated a role of CD14 in platelets. *Wang et al.* demonstrated that human megakaryocytes express CD14^24^, suggesting that platelets may contain CD14 molecules synthesized by their precursor cells. In addition, other investigations confirmed the presence and the functional activity of the intracellular isotype forms TLR7 and TLR9 in platelets^4–8,25^, as isotypes in immune cells typically requiring CD14 as constitutive co-receptor for the recognition and absorption of nucleic acids^26^. In studies using proteomic analysis, the presence of CD14 molecules in human platelets has not been reported, however its identification has not been specifically addressed^27^.

In addition, it is important to evaluate the distribution of CD14 in subcellular compartments since functional or pathophysiological processes may be associated with dislocation of CD14. Transmission electron microscopy of quiescent suspended platelets showed a specific intracellular staining of CD14, predominantly localized along the membrane and outside of granules. The approximate correction of deviation of the gold particles bound to the anti-CD14 antibody suggest that CD14 molecules may be located in the open canalicular system.

Surprisingly, the visualization of adherent and collagen-activated, non-permeabilized platelets using immunofluorescence microscopy revealed a locally condensed surface expression of CD14. Upon activation and adherence on collagen-coated surfaces, platelets undergo changes of their structural organization resulting in centralization of cytoskeletal elements and organelles including mitochondria, α-granules and dense granules, thereby transferred to close association with the open canalicular system^20^. CD14 expression was also inducible under stimulation of platelets with convulxin, pointing to the potential involvement of the immune receptor GPVI in recruitment mechanisms for CD14^28,29^.

Furthermore, the number and the size of the open canalicular system increase during platelet activation, promoting platelet secretion^20^. Therefore, it may be assumed that enlarged apertures of the open canalicular system in activated adherent platelets enable the access of anti-CD14 antibody to intracellular CD14 molecules for staining.

In permeabilized platelets, a thread-like CD14 staining pattern was observed along the intracellular membrane and in peripheral sections, in addition to the locally concentrated fluorescent clusters as observed in non-permeabilized platelets. These thread-like structures are in accordance with the localization of CD14 in the microtubular system and in the open canalicular system^20^.

As a limitation of this study, it should be mentioned that this study focused on general CD14 expression in platelets. CD14 may also be relevant for dimerization of other molecules like TLR4/MD-2^15^. In investigations with LPS-induced effects on platelet secretion, it was suggested that sCD14 from plasma contributes to LPS/TLR4 signaling^30^, which cannot be completely ruled out, since under in vivo-conditions platelets are permanently in contact with sCD14 in the blood stream. In next studies, therefore, it will be mandatory to address the functional role of CD14 and of sCD14 in different mechanisms of platelet activation and in signaling, e.g. in the TLR4-MyD88 receptor-signaling complex required for LPS signaling, which is also present in human platelets and involved in platelet secretion^31^. Furthermore, it will be important to address the functional role of CD14 in platelets, since dysfunction of platelets may be associated with CD14-dependent mechanisms.

Immunofluorescence microscopy of adherent platelets on collagen-coated surfaces indicated that CD14 is present in co-localization with TLR2 and TLR4. In classical immune cells, CD14 serves as an essential co-receptor of various TLR and is usually involved in the uptake and translocation of ligands into the cell^26,32,33^. In this regard, it is remarkable that stimulation of TLR2 induces an uptake of fibrinogen and Pam3CSK4^17^, whereas such effects are not mediated via TLR4 stimulation with LPS. It may be suggested that Pam3CSK4-induced CD14 expression on the platelet surface is required to promote the internalization of surrounding small molecules and TLR2-bound ligands, facilitating their intracellular inactivation with ROS^17^. Interestingly, it was shown that TLR2- and TLR4-mediated responses were suppressed in the milieu of platelet-rich plasma^22^. It would be of interest to analyze if CD14 may be involved in this phenomenon, e.g. by blocking CD14 during TLR activation. In the clinical context, the occurrence of adverse reactions after the transfusion of platelet concentrates is potentially attributable to overshooting TLR-mediated responses^4^. Therefore, CD14 may also be considered as a potential target to interrupt TLR-dependent transfusion reactions.

For this study, it was essential to use a multi-modal, comprehensive approach including different methods to provide evidence for CD14 in human platelets. Single experiments of each method were repeatedly performed (each single experiment with material from different donors), indicating equal results and supporting reliable conclusions.

**Fig. 8:**
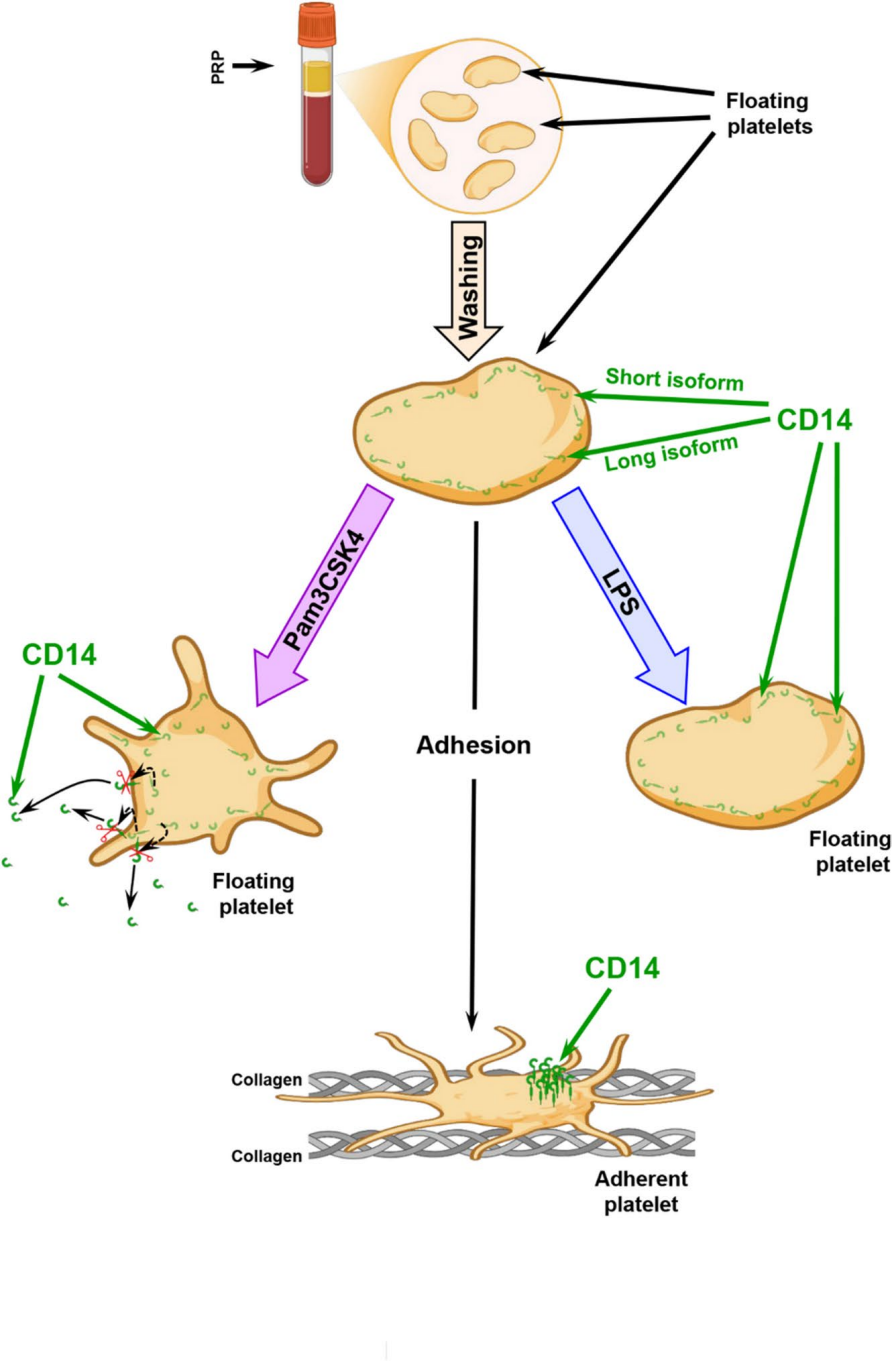
Mechanism of CD14 regulation in human platelets. Collagen-adherent platelets express CD14 on their surface and TLR2-stimulated platelets release sCD14. The figure was created with the software BioRender (URL: https://biorender.com, version used on 2025-05-30; Created in BioRender. Kobsar, A. (2025) https://BioRender.com/ammeo3s).

In summary, it has become evident that platelets contain CD14 molecules, located along the intracellular membrane (Fig. 8). Upon specific stimulation, e.g. via TLR2 or collagen, but not via TLR4, CD14 is expressed on the surface membrane and partially released as sCD14 into the surrounding milieu. Activated platelets, adherent to collagen-coated slides, show CD14 expression in a limited area close to the “yolk” spot. The specific regulation of CD14 expression and CD14 release may have an important role for the induction of specific responses to different platelet stimuli, e.g. via TLR2 or TLR4, which are co-localized with CD14.

## Materials and methods

### Materials

The synthetic triacylated lipopeptide Pam3CysSerLys4 VacciGrade (Pam3CSK4) was obtained from InvivoGen (Toulouse, France). Lipopolysaccharide (LPS) from Escherichia coli O111:B4, thrombin, Ethylene glycol-bis(β-aminoethylether)-N, N,N′,N′-tetraacetic acid (EGTA), prostaglandin E1 (PGE1), Ponceau S, fluorescein isothiocyanate (FITC)-conjugated rabbit anti-goat antibody (Sigma-Aldrich Cat# F2016, RRID: AB_259459) and rabbit anti-CD14 antibody (Sigma-Aldrich Cat# HPA001887, RRID: AB_1078442) were from Sigma-Aldrich Chemie/Merck (Muenchen, Germany). The polyclonal goat anti-CD14 antibody (Novus Cat# NB100-2807, RRID: AB_10002272) and the isotype control goat IgG (Novus Cat# NB410-28088, RRID: AB_1853319) were from Bio-Techne (Minneapolis, USA). Mouse anti-TLR2, mouse anti-TLR4, mouse IgG2a, Alexa Fluor Plus 488-conjugated donkey anti-goat antibody, Dylight 550-conjugated donkey anti-mouse antibody (Thermo Fisher Scientific Cat# 17–9922-42, RRID: AB_11042560; Cat# 16–9917-82, RRID: AB_469280; Cat# MA1-10418, RRID: AB_2536786; Cat# A32814, RRID: AB_2762838; Cat# SA5-10167, RRID: AB_2556747) were from Thermo Fisher Scientific (Waltham, USA), human VioBlue-conjugated anti-CD41a antibody (Miltenyi Biotec Cat# 130-123-876, RRID: AB_2819532) was from Miltenyi Biotec V.B. & Co.KG (Bergisch Gladbach, Germany). Horseradish peroxidase (HRP)-conjugated rabbit anti-goat antibody (Bio-Rad Cat# 1721034, RRID: AB_2617114) was from Bio-Rad Laboratories, Inc. (Muenchen, Germany). The antibody goat IgG anti-rabbit IgG (H + L)-Gold 12 nm (Jackson ImmunoResearch Labs Cat# 111–205-144, RRID: AB 2338016) was from Jackson ImmunoResearch (Ely, Cambridgeshire, United Kingdom). Collagen Reagens HORM was from Takeda (Linz, Austria). Convulxin (37206-04-5) was from Cayman Chemical (Michigan, USA). Quantikine QuicKit ELISA CD14 Immunoassay was from R&D Systems GmbH (Wiesbaden-Nordenstadt, Germany).

### Blood collection and Preparation of WP

Our studies with human platelets and the consent procedure were approved by our local ethics committee of the University of Wuerzburg (approval number 101/15). The participants provided their written informed consent to participate in this study. The study was performed according to our institutional guidelines and to the Declaration of Helsinki.

Venous whole blood samples were obtained from healthy voluntary donors without any medication intake. Peripheral blood was collected in four polypropylene tubes containing 3.2% citrate buffer (106 mM trisodium citrate, Sarstedt, Nuembrecht, Germany). For the preparation of WP, 3 mM EGTA was added to whole blood samples to prevent platelet activation. Platelet-rich plasma (PRP) was obtained from whole blood by centrifugation at 280 g for 5 min. Only the upper part of PRP (approximately 3/4) over the red blood cell layer was used for platelet preparation to avoid leukocyte contaminations. This upper part of PRP was transferred into a new tube and platelets were pelleted by 5 min centrifugation at 430 g. After that, pelleted platelets were washed once with CGS buffer (120 mM sodium **c**hloride, 30 mM D-glucose, 12.9 mM trisodium citrate, pH 6.5) and re-suspended in HEPES buffer (10 mM HEPES, 10 mM D-Glucose, 150 mM NaCl, 5 mM KCl, 1 mM MgCl2, pH 7.4) to the appropriate concentration. After resting for 15 min at 37 °C in a water bath, WP were used for further experimentation.

Blood cell count in samples was performed with a Sysmex KX-21 N haematology analyzer (Sysmex Europe GmbH, Norderstedt, Germany).

### Preparation of WM

For the preparation of WM, the lower part of PRP (approximately 1/4) over the red blood cell layer and approximately 1 mL of erythrocytes were collected in a separate tube and carefully mixed. Afterwards 5 ml of the cell mixture were carefully layered over 5 ml Polymorphoprep and centrifuged for 35 min at 550 g. After that, the upper leucocyte band, containing mononuclear cells, was harvested. Consecutively, monocytes were washed twice with PBS (430 g for 10 min) to remove residual Polymorphoprep before re-suspension in HEPES buffer adjusted to 1 × 10^7^ cells per mL.

### Detection of CD14 expression on the platelet surface

For the detection of TLR2- or TLR4-stimulated CD14 expression, 50 µL of WP were adjusted to 3 × 10^8^ platelets per mL, supplemented with 1 mM CaCl_2_ and pre-incubated with 1 µL of goat anti-CD14 antibody or goat IgG (isotype control) for 10 min at 37 °C. In the next step, samples were stimulated with buffer (HEPES), 15 µg/mL LPS or 15 µg/mL LPS for 5 min at 37 °C, and fixed with 1% formaldehyde for 10 min at RT. Consecutively, the samples were centrifuged for 1 min at 20,000 g, and the pellets were re-suspended in 100 µL of PBS/BSA/Glc (Dulbecco’s PBS Ca2+, Mg2+-free, 0.5% bovine serum albumin, 5.5 mM D-glucose) and stained with 0.5 µL of FITC-conjugated rabbit anti-goat antibody for 30 min at RT. Finally, all samples were diluted with 700 µL of PBS/BSA/Glc buffer and analyzed by flow cytometry using a FACS Calibur flow cytometer from Becton Dickinson (Franklin Lakes, NJ, USA) and the CELLQuest software, version 6.0. The platelet population was identified by its forward and side scatter distribution and the fluorescence values of 10,000 events were analyzed.

For detection of convulxin-stimulated CD14 expression, two tubes with 300 µL of WP suspension adjusted to 3 × 10^8^ platelets per mL, supplemented with 1 mM CaCl_2_ and pre-incubated for 10 min at 37 °C were stimulated with buffer (HEPES) or 100 ng/mL convulxin. After stimulation for 1, 3, 5, 10 and 30 min, 50 µL of platelet suspension were taken from the stimulated WP mixture and fixed in separate tubes with 5 µL of 10% formaldehyde (1% final concentration) for 10 min at RT, followed by centrifugation at 20,000 g for 1 min. The pellets were re-suspended in 100 µL of PBS/BSA/Glc and divided into two tubes with 50 µL WP in each. One of them was stained with 0.5 µL of goat anti-CD14 antibody, and another with isotype IgG for 30 min at RT, followed by a second centrifugation and staining with 0.5 µL of FITC-conjugated rabbit anti-goat antibody in 50 µL of PBS/BSA/Glc for 30 min at RT. Finally, samples were diluted with 700 µL of PBS/BSA/Glc and analyzed by flow cytometry as described above.

### Measurement of soluble CD14 (sCD14) secretion

200 µL of WP, prepared as described above, were adjusted to 1 × 10^9^ platelets per mL, supplemented with 1 mM CaCl_2_ and stimulated with buffer (HEPES), 15 µg/mL Pam3CSK4 or 15 µg/mL LPS for 30 min at 37 °C. After that, 2 µL of 0.5 M EGTA was added to each sample to prevent platelet aggregation before centrifugation for 5 min at 430 g. The supernatants were collected in new tubes and frozen at −80 °C for measurement of secreted soluble CD14 with the Quantikine QuicKit ELISA CD14 Immunoassay. As a positive control, 200 µL of WM (1 × 10^7^ cells per mL) were used.

### Western blot analysis of CD14 expression

The content of CD14 in platelets was determined by Western blot analysis, as previously described^16^. Briefly, prepared WP pellets and WM pellets were re-suspended in 200 µL of HEPES buffer and immediately lysed with 100 µL of Laemmli buffer (200 mM Tris, 15% (v/v) glycerol, 6% (w/v) SDS, 0.3% (w/v) bromphenolblue, 10% (v/v) 2-mercaptoethanol, pH 6.7). The cell lysates were loaded onto the gel, separated by SDS-PAGE and then transferred onto nitrocellulose membranes. Ponceau S staining was used for loading control and normalization^34^. The membranes were incubated with goat anti-CD14 antibodies (1:100 dilution) overnight at 4 °C. For visualization of the signal, a HRP-conjugated secondary antibody was used, followed by detection with Chemidoc MP imaging system (Bio-Rad Laboratories, Inc., Hercules, CA, USA) and analysis with the corresponding Image Lab software, version 6.0.

### Transmission electron microscopy of WP

The localization of intracellular CD14 in quiescent WP was determined by transmission electron microscopy. For this purpose, 1,200 µL of WP suspension (1 × 10^9^ platelets per mL) were fixed with 400 µL of 16% freshly prepared paraformaldehyde in PBS (4% final concentration) for 2 h on ice. Fixed platelets were pelleted by centrifugation at 430 g for 10 min. The pellet was carefully washed three times for 3 min with cold PBS. After saturation of free aldehyde groups with 50 mM NH_4_Cl in PBS for 15 min at RT and followed by washing with water five times for 3 min, platelet pellet was dehydrated by an ethanol series and embedded with LR white resin according to Table 1.

**Table 1:** Experimental steps for dehydration of platelet pellet.

30% Ethanol	2 × 15 min	4 °C
50% Ethanol	2 × 30 min	− 20 °C
70% Ethanol 0.2% Uranyl Acetate	2 × 30 min	− 20 °C
90% Ethanol 0.2% Uranyl Acetate	2 × 30 min	− 20 °C
100% Ethanol	1 × 60 min	− 20 °C
100% Ethanol	Overnight	− 20 °C
100% Ethanol/LR-White 1:1	Overnight	4 °C
LR-White	1 h	4 °C
LR-White	3–4 h	4 °C
LR-White	Overnight	4 °C
LR-White	3–4 h	22 °C

After that, the preparations were transferred into gelatine capsules, closed and polymerized at 42 °C in an oven for at least 3 days.

The LR-White embedded platelets were cut with an ultra-microtome (Leica EM UC7, Leica Microsystems, Wetzlar, Germany) to approximately 70 nm thin slices, which were placed on the carbon-coated nickel 100 mesh grids and dried overnight. The mesh bound slices were washed in PBS drop for 5 min at RT, followed by 5 min blocking with 1% BSA/0.1% Tween-20 in PBS. After that, the mesh bound slices were stained with rabbit anti-CD14 antibody (1:50) in a humid chamber for 1 h at RT. The primary antibody was washed off with 1% BSA/0.1% Tween-20 in PBS (three times for 10 min at RT) and with 0.1% BSA/0.1% Tween-20 in PBS (twice for 10 min at RT). Staining with secondary goat-anti-rabbit 12 nm colloidal gold-conjugated antibody was performed in 0.1% BSA/0.1% Tween-20 (1:10) for 1 h at RT. After washing with 0.1% BSA/0.1% Tween-20 in PBS (three times for 10 min at RT), 2 min fixation with 1.25% glutaraldehyde in PBS and final washing with double distilled water (three times for 5 min), the mesh bound slices were contrasted for 5 min with aqueous 2% uranyl acetate solution. For contrast enhancement, the mesh slices were also treated with Reynolds’ lead citrate solution^35^. The slides were imaged in the Imaging Core Facility at the Theodor-Boveri-Institute of Bioscience (University Wuerzburg, Germany) with the help of transmission electron microscope JEOL JEM-1400Flash (JEOL Germany, Freising, Germany) equipped with a Matataki camera (JEOL Germany, Freising, Germany) at 120 kV.

### Immunofluorescence staining

8-wells slide was coated with collagen (1 µg/cm^2^) for 60 min at RT and washed with PBS. 240 µl of WP suspension adjusted to 2.5 × 10^7^ platelets/mL were seeded in each well of the slide for 30 min at RT. After that, platelets were fixed with 80 µL of 16% paraformaldehyde (PFA) in PBS (4% final PFA concentration) for 10 min on ice. After a washing step with PBS, the platelets in 4 from 8 wells were permeabilized for 10 min at RT with 0.2% Triton X-100 in PBS. The samples were blocked with 1% BSA in PBS with 0.2% Triton X-100 for permeabilized platelets or without for non-permeabilized platelets for 1 h at RT and subsequently stained with VioBlue-conjugated human anti-CD41a (1:100), mouse IgG2a or anti-TLR2 antibody or anti-TLR4 antibody (1:50 dilution) and goat IgG as isotype control or goat anti-CD14 antibody (1:100) diluted in 1% BSA in PBS overnight at 40 °C. Next day, the slide was washed five times with PBS and stained with Dylight 550-conjugated donkey anti-mouse antibody (1:100 dilution) and Alexa Fluor Plus 488-conjugated donkey anti-goat antibody (1:100) diluted in 1% BSA in PBS for 1 h at RT, followed by five times washing with PBS. Finally, the slide was mounted with ProLong Diamond Antifade Mountant (Invitrogen, Thermofisher Scientific, Eugene, USA). After 24 h of curing at RT, the slide was stored at 40 °C until immunofluorescence imaging. Mounted samples were imaged on an inverted Nikon Eclipse Ti2 microscope (Nikon GmbH, Duesseldorf, Germany) using a 100 × oil immersion objective and 1.5x Zoom (14-bit digitalization). For improved figure presentation, the dark blue florescence of VioBlue-conjugate was changed to red in case of sole CD14 staining or to cyan in case of co-localization staining by means of ImageJ 1.53f51 program (NIH, USA).

### Statistical analysis

Descriptive data were calculated with GraphPad PRISM 7 (GraphPad Software, San Diego, CA, USA). Data distribution analysis was performed with Shapiro-Wilk test. Differences of variances between groups were analyzed by one-way analysis of variance (ANOVA) or by paired Student’s t test as appropriate. *P* < 0.05 was considered statistically significant.

## Supplementary Information

Below is the link to the electronic supplementary material.

Supplementary Material 1

Supplementary Material 2

Supplementary Material 3

Supplementary Material 4

Supplementary Material 5

## Data Availability

All data generated or analysed during this study are included in this published article (and its Supplementary Information files) or available from the corresponding author on reasonable request.
