# Supplementary material for: TLR2-induced surface mobilization and release of CD14 in human platelets
Source: Sci Rep. 2025 Oct 13;15:35572. doi: 10.1038/s41598-025-22715-7 (PMC12518645; doi:10.1038/s41598-025-22715-7)
Supplement: Supplementary file 1 — Supplementary Material 1 [file 41598_2025_22715_MOESM1_ESM.pdf]

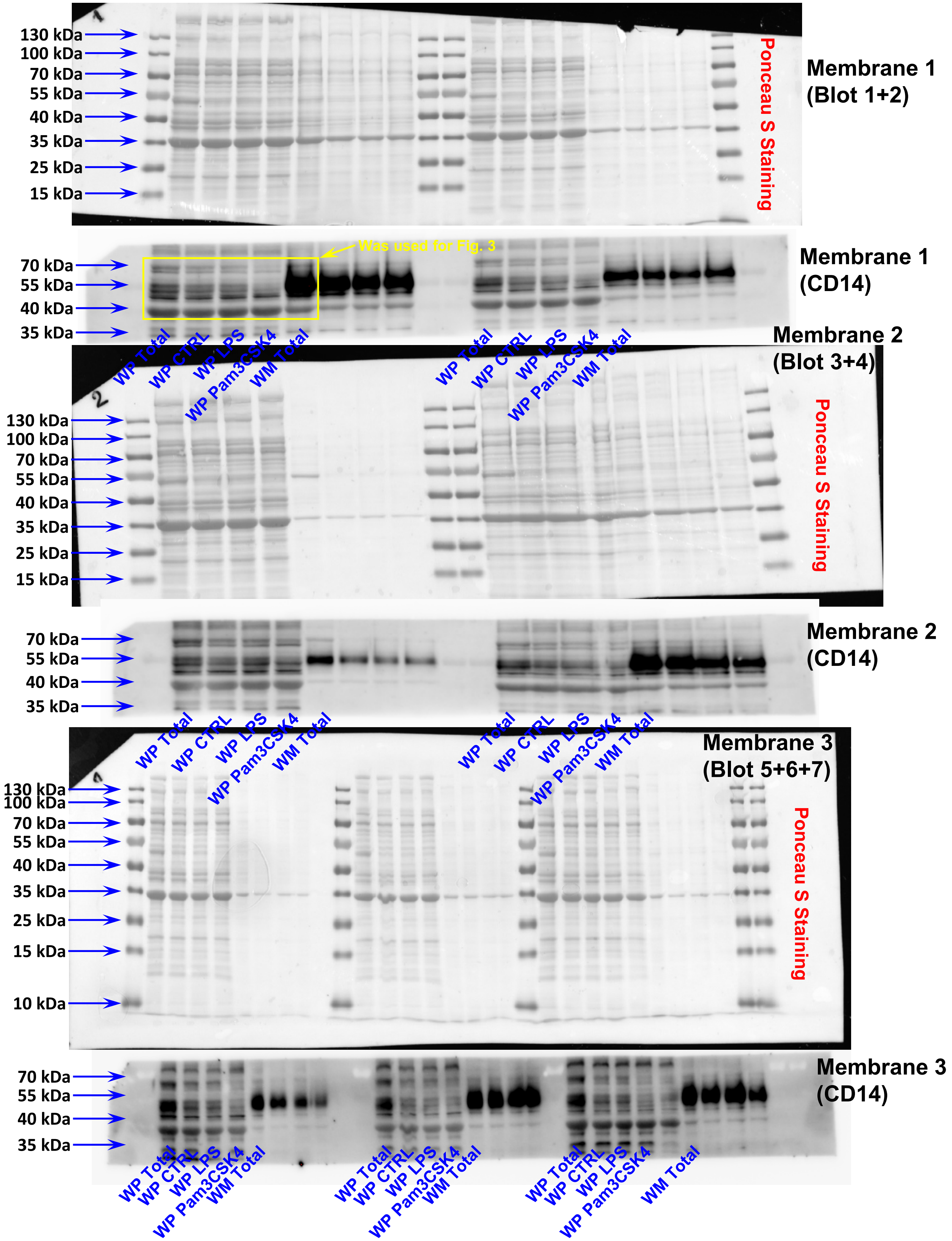


**Figure S1:** The figure shows uncropped membranes of Western Blot analysis illustrated in figure 3. CD14 was detected in lysed pellets of washed platelets (WP) after incubation with buffer (CTRL, control), 15 µg/mL LPS or 15 µg/mL Pam3CSK4 for 30 min (using a goat anti-CD14 primary antibody and a HRP-conjugated secondary antibody). Washed human monocytes (WM) served as positive controls. Ponceau S staining was used as loading control; n=7.
