# Supplementary material for: TLR2-induced surface mobilization and release of CD14 in human platelets
Source: Sci Rep. 2025 Oct 13;15:35572. doi: 10.1038/s41598-025-22715-7 (PMC12518645; doi:10.1038/s41598-025-22715-7)
Supplement: Supplementary file 4 — Supplementary Material 4 [file 41598_2025_22715_MOESM4_ESM.pdf]

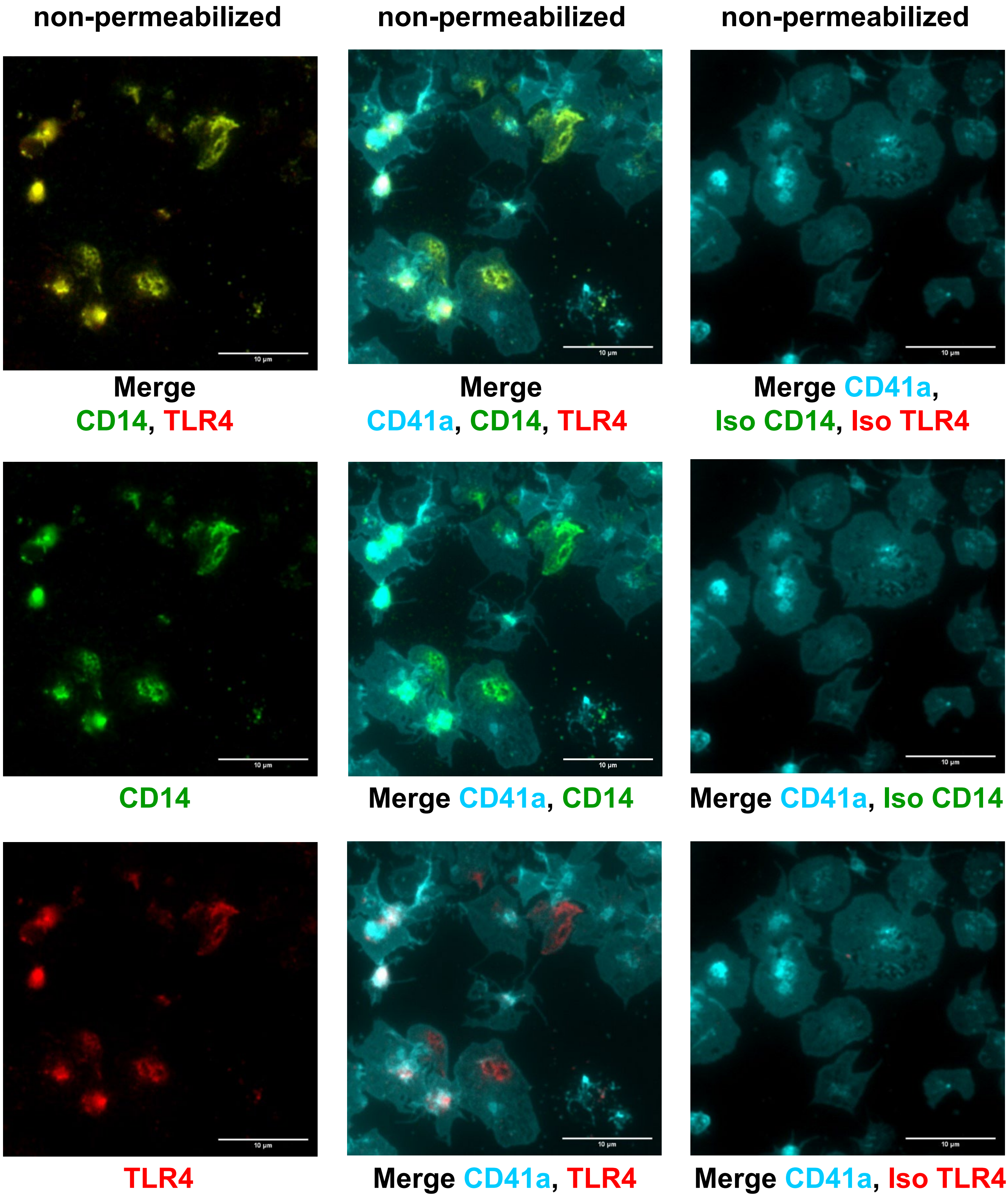

Figure S4: Co-localization of CD14 with TLR4 in adherent, non-permeabilized platelets is shown. Washed platelets (2.5x 10^7 /mL), diluted with HEPES and supplemented with 1 mM $CaCl_2$, were seeded onto collagen-coated slides and left to adhere for 30 min at room temperature. After fixation, platelets were stained with goat anti-CD14, mouse anti-TLR4 and Vioblue-conjugated anti-CD41a antibodies or adequate isotype controls overnight as indicated, followed by staining with Alexa Fluor 488-conjugated donkey anti-goat- and DyLight 550-conjugated donkey anti-mouse antibodies, mounting and subsequent analysis on an inverted Nikon Eclipse Ti2 microscope using a 100 x oil immersion objective and a 1.5x zoom (14-bit digitalization). The images show representative slides with adherent unstimulated platelets; n=5.
